# Unconstrained Coordination of Pt Single Atoms on Distorted g‐C_3_N_4_ Enables Electronic Flexibility and Enhanced Microplastics Photoreforming

**DOI:** 10.1002/advs.76070

**Published:** 2026-07-29

**Authors:** Ting‐Han Lin, Yin‐Hsuan Chang, Jia‐Mao Chang, Ciao‐Yun Huang, Kai‐Chi Hsiao, Kuo‐Ping Chiang, Ying‐Han Liao, Kai‐Hsiang Hsu, Jen‐Fu Hsu, Ming‐Chung Wu

**Affiliations:** ^1^ Center for Sustainability and Energy Technologies Chang Gung University Taoyuan Taiwan; ^2^ Department of Chemical and Materials Engineering College of Engineering Chang Gung University Taoyuan Taiwan; ^3^ Division of Neonatology Department of Pediatrics Chang Gung Memorial Hospital at Linkou Taoyuan Taiwan; ^4^ Graduate Institute of Clinical Medical Sciences College of Medicine Chang Gung University Taoyuan Taiwan; ^5^ School of Medicine College of Medicine Chang Gung University Taoyuan Taiwan; ^6^ Center for Heterogeneous and Innovative Post‐Silicon Materials Chang Gung University Taoyuan Taiwan; ^7^ Department of Materials Engineering Ming Chi University of Technology New Taipei City Taiwan

**Keywords:** graphitic carbon nitride, photoreforming, plastic‐to‐hydrogen conversion, single‐atom catalysts

## Abstract

The accumulation of microplastic waste has raised growing environmental concerns, motivating the development of sustainable strategies for plastic‐to‐fuel conversion. Here, we report a platinum single‐atom catalyst anchored on distorted graphitic carbon nitride (Pt_1_/CN) for efficient photocatalytic microplastics photoreforming. Unlike conventional planar g‐C_3_N_4_ models, the locally distorted heptazine framework introduces off‐plane coordination environments that enable unconstrained Pt─N coordination and promote electronic flexibility. X‐ray absorption spectroscopy reveals Pt─N coordination associated with distorted heptazine units, where distortion‐induced charge localization enhances interfacial electron transfer to Pt sites. In situ measurements under light irradiation further confirm efficient charge transfer at the Pt_1_/CN interface. Among various microplastics, polyethylene terephthalate (PET) exhibits the highest hydrogen evolution rate of 533.18 µmol·g^−1^·h^−1^, attributed to alkaline‐assisted ester bond cleavage. Density functional theory calculations demonstrate that Pt single atoms facilitate hydrogen evolution by lowering H^+^ reaction barrier and stabilizing key intermediates. This work elucidates the structure–activity relationship of Pt_1_ in polymeric semiconductors and establishes a framework‐level design strategy for electronic flexibility in photocatalytic plastic‐to‐fuel conversion.

## Graphical abstract

This study reveals Pt single atoms anchored on distorted graphitic carbon nitride, exhibiting flexible coordination and enhanced interfacial charge transfer. Combined spectroscopy and DFT reveal asymmetric Pt─N environments that promote photoreforming of microplastics into hydrogen, demonstrating a solar‐driven pathway for plastic upcycling with experimentally validation and practical reactor feasibility.

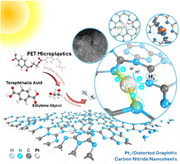

## 1 Introduction

The accelerating crisis of plastic pollution, primarily driven by the disposal of plastic packaging, represents both an environmental threat and a significant waste of valuable resources. Approximately 86% of plastic packaging finds its way into landfills, leading to a considerable loss of resources, especially considering the fossil fuel origin of most plastics [1]. The widespread adoption of recycling initiatives faces obstacles due to inefficient waste management systems, a lack of public awareness, and the varied chemical compositions, complexities, and sizes of plastic products. Particularly problematic in the recycling effort are microplastics. The tiny plastic particles less than 5 mm in size infiltrating hydrological ecosystems [2] present formidable challenges in their collection and subsequent reuse, posing grave risks to both environmental and human health [3]. Advanced solutions for plastic waste management are essential to not only address the efficient processing of plastic waste but also transform it into valuable new materials, paving the way for a more sustainable approach to dealing with the plastic pollution crisis.

Advancements in catalytic technologies, such as electrocatalysis [4, 5, 6], thermocatalysis [7], photothermal catalysis [8], and photocatalysis [9, 10, 11, 12, 13], are complementary treatment options to traditional waste disposal methods like landfilling, incineration, and mechanical recycling. Photocatalysis exhibits distinct capability in the fields of sustainable waste management and renewable energy generation [14, 15, 16, 17]. A key breakthrough in this area is photoreforming, which has emerged as a promising technique for transforming plastic waste into hydrogen fuel using sunlight. A representative photoreforming reaction can be summarized by the following equation [18, 19]:

$$C_{x}H_{y}O_{z}+\left(2x-z\right)H_{2}O\to \left(2x+(y/2)-z\right)H_{2}+\mathrm{xC}O_{2}$$

Its merit lies in its sequential functionality: plastic decomposition into organic substrates via hydrolysis under mild alkaline conditions, followed by proton (H^+^) reduction to hydrogen under catalytic conditions [20, 21, 22]. This process effectively bypasses the kinetically demanding water oxidation reaction by employing organic substrates as sacrificial electron donors, thus facilitating hydrogen evolution in a photocatalytic system. Currently, photoreforming of plastics to hydrogen remains largely at the fundamental research stage. Its practical implementation requires breakthroughs in system integration, and particularly in the applicability of the catalyst [23]. Hence, with an in‐depth mechanistic understanding and precise atomic‐level control of active sites, catalysts can be effectively tailored to enable tunable reaction dynamics for efficient H_2_ evolution.

Graphitic carbon nitride (g‐C_3_N_4_) stands out as a promising semiconductor since the groundbreaking research conducted by Antonietti's team in 2009, which demonstrated a key example in light‐driven water splitting [24]. Owing to its moderate bandgap of 2.7–2.8 eV, and electronic band positions that meet the thermodynamic requirements for hydrogen evolution, g‐C_3_N_4_ has been widely used in solar‐driven catalysis [25, 26, 27]. Its unique conjugated polymeric system composed of tri‐s‐triazine (heptazine) units, and 2D nanosheet offers structural accessibility for loading or anchoring active species to achieve enhanced catalytic performance [28, 29, 30]. Atomic‐level engineering first demonstrated that atomically dispersed platinum atoms (Pt_1_) on metal oxides can achieve efficient CO oxidation [31], thereby opening a new avenue in heterogeneous catalysis [32, 33, 34]. In this context, Wei et al. reported that isolated Pt and Pd atoms can be stabilized on g‐C_3_N_4_, functioning as active sites for visible‐light‐driven CO_2_ reduction. The anchoring of single atoms posed a promising strategy to achieve high catalytic efficiency with minimal cocatalyst loading of precious metals [35]. Yang et al. demonstrated Pt SAC on g‐C_3_N_4_ with an ultra‐low Pt loading, and highlighted the importance of balancing catalytic activity with the efficient use of precious metals. A pronounced two‐electron oxygen reduction pathway was facilitated by Pt atoms [36]. Lazaar et al. reported the low loadings of Pt SAC (< typically 2.0–5.0 wt.%), maximizing hydrogen evolution reaction (HER) efficiency via self‐guiding of Pt‐acid precursor in most active surface configurations [37]. This report suggests the significance of well‐defined Pt atom coordination in g‐C_3_N_4_.

To further elucidate the role of these coordination structures during actual catalytic processes, subsequent investigations have focused on reaction intermediates, selectivity, and electronic modulation. Hu et al. achieved efficient CO selectivity in CO_2_ photoreduction using Pt SAC anchored on porous C_3_N_4_. That unveiled a strong correlation between key reaction intermediates and Pt─N_4_ coordination, which served as active sites during the dynamic CO_2_ conversion process [38]. Yadav et al. found that the Pt SACs coordinate and interact with the N‐rich sites, altering the electronic structure of the N‐rich C_3_N_4_ [39] Pt atoms deposited on g‐C_3_N_4_ surfaces via conventional methods often exhibit random distribution due to the absence of well‐defined active sites and coordination environments. This uncertainty in spatial arrangement highlights the critical role of controlled Pt SAC distribution and coordination structure within g‐C_3_N_4_, which fundamentally governs catalytic performance. As a consequence, the flexibility of g‐C_3_N_4_ increases the surface energy, often resulting in the random distribution of single atoms across its layered structure. Sophisticated characterizations such as STEM‐HAADF, XANES, and EXAFS with wavelet analysis offer unprecedented insights into atomic‐scale architectures and structural information. However, prevailing DFT models and EXAFS interpretations remain predominantly anchored to idealized, planar g‐C_3_N_4_ templates. This reliance on simplified geometries systematically neglects the role of structural distortion in modulating Pt coordination [40, 41, 42, 43]. In reality, the native form of g‐C_3_N_4_ more closely resembles flexible, distorted nanosheets with interlayer deviations from planarity [44]. These structural distortions including changes in stacking order, atomic distances, and bonding angles can significantly influence the local coordination geometry and electronic properties of Pt single atoms [45]. DFT calculations based on planar models diverge from actual experimental conditions, potentially leading to misinterpretations of charge transfer dynamics and catalytic behavior. This highlights the need for structural modeling with reliable experimental validation that accounts for nanosheet distortion to more accurately guide the rational design and optimization of SAC/g‐C_3_N_4_ for improved catalytic performance.

Herein, we demonstrate Pt single‐atom catalysts anchored on distorted g‐C_3_N_4_ nanosheets (Pt_1_/C_3_N_4_) for the photoreforming of microplastics to hydrogen and elucidate the local coordination environment of Pt single atoms through an integrated experimental‐theoretical approach. Advanced spectroscopic characterizations combined with DFT modeling reveal that asymmetric Pt─N coordination induces interfacial charge redistribution and enhances proton reduction activity. Photoreforming tests using representative microplastics identify PET as the most active substrate, delivering a high hydrogen evolution rate. Furthermore, a realistically designed photoreforming reactor demonstrates the practical feasibility of this strategy under continuous operation.

## 2 Results and Discussion

**FIGURE 1:**
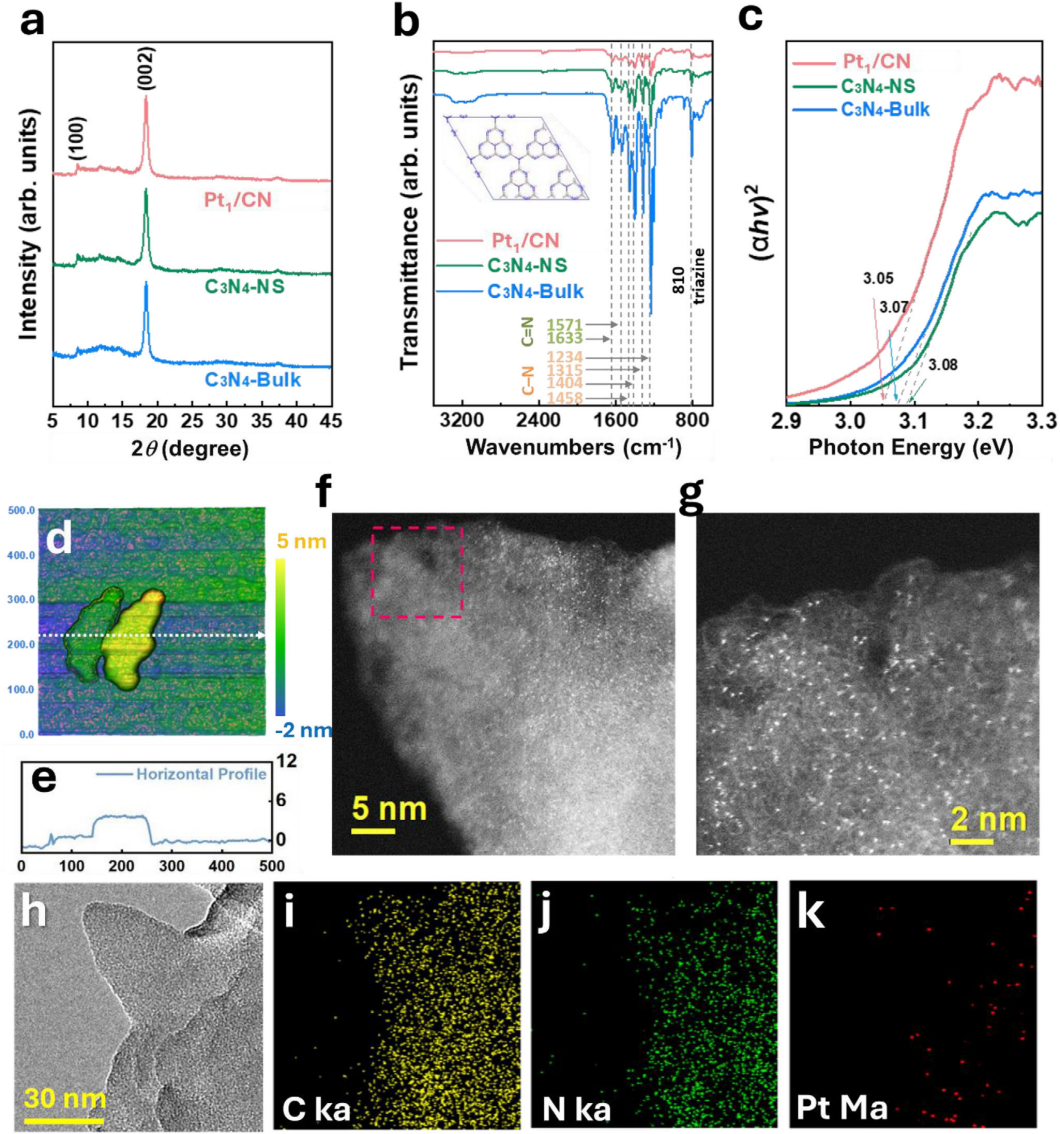
Characterization of crystal structure and microstructure: (a) synchrotron x‐ray spectra, (b) ATR‐FTIR patterns, (c) Tauc plot of C_3_N_4_‐Bulk, C_3_N_4_‐NS, and Pt_1_/CN. Morphology of single atomic Pt_1_/CN. (d, e) AFM height image line scan and corresponding height profile. (f) HAADF‐STEM image, (g) magnified HAADF‐STEM image highlighting atomically dispersed Pt species, and (h) TEM image with elemental mapping of (i) carbon, (j) nitrogen, and (k) platinum.

The key limitation of bulk g‐C_3_N_4_ arises less from the absence of stacking order than from over‐stacking and macroscopic aggregation, which hinder interfacial reactions and promote charge‐carrier recombination. To overcome the intrinsic limitations, we developed a facile strategy to synthesize Pt single atoms anchored on g‐C_3_N_4_ (Pt_1_/CN) using urea and platinum chloride as the precursors, followed by ultrasonic exfoliation to obtain ultrathin nanosheets. The ultrasonic treatment effectively disassembled the stacked layers, resulting in an increased specific surface area, and accessible surface for reactions. The morphologies in Figure S1 show that C_3_N_4_‐Bulk consists of relatively thick, stacked sheets, while C_3_N_4_‐NS exhibits a more delaminated and flakier morphology, confirming the successful bulk‐to‐nanosheet transformation after exfoliation. The textural properties of C_3_N_4_‐Bulk and C_3_N_4_‐NS were investigated by N_2_ adsorption‐desorption isotherm measurements, shown in Figure S2 and Table S1. According to IUPAC classification, both samples show type IV isotherms with H_3_ hysteresis loops, suggesting the existence of slit‐like pore characteristic of mesoporous materials. The BET specific surface area of C_3_N_4_‐Bulk and C_3_N_4_‐NS samples are 72.28 and 77.77 m^2^·g^−1^, respectively. Pore volume increased from 0.42 to 0.51 cm^3^·g^−1^, and pore size increased from 23.21 to 24.30 nm. Synchrotron x‐ray diffraction was employed to elucidate the phase structural transformations among C_3_N_4_‐Bulk, C_3_N_4_‐NS, and Pt_1_/CN (Figure 1a). All samples exhibited a characteristic diffraction peak at 18.3°, corresponding to the (002) plane, which represents the interplanar long‐range stacking of g‐C_3_N_4_ aromatic structures. The weak peak at 8.5°, corresponding to the (100) plane, is also attributed to the in‐plane repeating motifs of the tri‐s‐triazine units. Compared to C_3_N_4_‐Bulk, the diffraction peaks for C_3_N_4_‐NS and Pt_1_/CN, particularly the (100) peak, are less distinct, suggesting a reduction in the planar dimensions. Meanwhile, the decreased intensity of (002) peak, relative to C_3_N_4_‐Bulk, implies a significant reduction of interlayer stacking strength, signifying a disintegration of the in‐plane network into thinner sheets. An unidentified impurity phase observed at 22.3° is absent following the Pt modification process. FT‐IR spectra of C_3_N_4_‐Bulk, C_3_N_4_‐NS, and Pt_1_/CN (Figure 1b) reveal that all three materials exhibit similar vibrational modes, with pronounced bands between 1100 and 1750 cm^−1^, certifying the preservation of the fundamental molecular framework of g‐C_3_N_4_ after the exfoliation and Pt decoration. The distinct peaks around 1234, 1315, 1404, and 1458 cm^−1^ are attributed to aromatic C─N stretch vibrations, while peaks at 1571 and 1633 cm^−1^ denote C═N stretching. Additionally, a prominent peak at 810 cm^−1^ is characteristic of the breathing mode of s‐triazine units, further confirming the structural integrity of the materials. UV–vis diffuse reflectance spectroscopy (DRS) was used to evaluate the influence of Pt incorporation on light absorption, and the optical bandgaps were estimated from Tauc plots (Figure 1c). Compared with C_3_N_4_‐Bulk, C_3_N_4_‐NS shows a similar absorption edge, whereas Pt_1_/CN exhibits a slight red shift and enhanced visible‐light absorption. The extracted bandgap energies are 3.07 eV (C_3_N_4_‐Bulk), 3.08 eV (C_3_N_4_‐NS), and 3.05 eV (Pt_1_/CN), suggesting a marginal narrowing upon Pt anchoring. The increased visible‐light absorption of Pt_1_/CN is attributed to Pt─g‐C_3_N_4_ electronic interactions that modify the local electronic structure and facilitate photoinduced charge transfer. The microstructures of Pt_1_/CN were characterized by atomic force microscopy (AFM) and high‐resolution transmission electron microscopy (HRTEM). As confirmed by AFM (Figure 1d,e), the thickness of Pt_1_/CN is ∼3.6 nm, which agrees with the formation of ultrathin nanosheet after exfoliation. The ultrasonically exfoliated Pt_1_/CN exhibits a layered structure, attributed to the disruption of interlayer van der Waals forces by intense ultrasonic vibrations, as shown in Figure 1f. The porous structure of Pt_1_/CN was formed with the released NH_3_ gas during the pyrolysis process of urea. HAADF‐STEM in Figure 1g further confirms atomic Pt (round and bright spots), showing that they are uniformly dispersed over the dark g‐C_3_N_4_ substrate. The compositional analysis was conducted through elemental mapping, as illustrated in Figure 1h–k, revealing the concurrent presence of carbon (C), nitrogen (N), and platinum (Pt) coexisted in the matrix. As depicted in Figure 1f,g, Pt_1_/CN demonstrated a homogeneous distribution of C and N across the specimens as well as a uniform distribution of Pt single atoms anchored on g‐C_3_N_4_.

**FIGURE 2:**
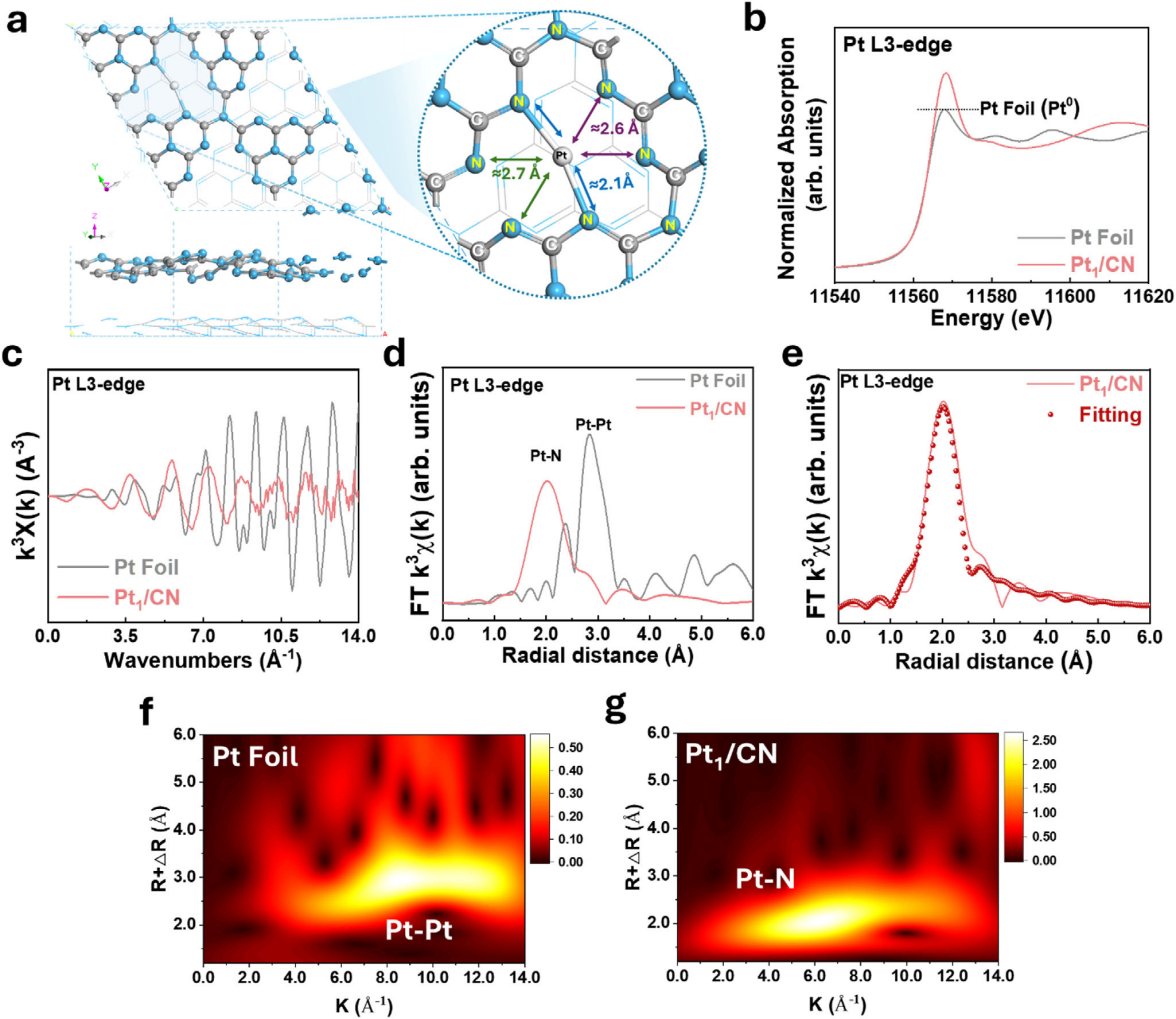
Illustration of chemical bond and XANES spectra of Pt on CN:(a) schematic illustration of the DFT‐optimized distorted structural model of Pt_1_/CN, showing the top view, cross‐sectional view, and magnified local coordination environment of a Pt single atom anchored at a heptazine unit. (b) Pt L_3_‐edge XANES spectra of Pt_1_/CN and Pt foil reference, along with the corresponding (c) *k*‐space EXAFS spectra and (d) Fourier‐transformed *R*‐space EXAFS spectra. (e) The fitted *R*‐space EXAFS spectrum of Pt_1_/CN using the optimized distorted structural model is also included. Wavelet‐transformed EXAFS spectra of (f) Pt foil and (g) Pt_1_/CN.

A combined theoretical‐spectroscopic analysis was conducted to elucidate the local coordination environment of Pt single atoms anchored on g‐C_3_N_4_. Under realistic synthesis conditions, g‐C_3_N_4_ nanosheets is inherently prone to structural distortion due to random thermal condensation, which induces nitrogen vacancies, disordered stacking, and poor crystallinity. As a result, the g‐C_3_N_4_ framework is better described as a flexible and nonplanar nanosheet rather than an idealized planar lattice. Figure 2a presents the DFT‐optimized distorted structural model of Pt_1_/CN, including the top view, cross‐sectional view, and a magnified local coordination site. In this model, the g‐C_3_N_4_ framework exhibits a laterally flexible, off‐plane distorted configuration, reflecting the intrinsic mechanical compliance of exfoliated g‐C_3_N_4_ nanosheets. Such off‐plane distortion, derived from ab initio calculations, has been shown to closely resemble the experimentally observed crystal structure of graphitic carbon nitride [27], and therefore, provides a more representative structural basis for describing the coordination environment of Pt single atoms. The DFT results also indicate that non‐equivalent Pt─N and Pt─C bond lengths are energetically favored, suggesting a more stable and asymmetric coordination geometry compared to idealized planar configurations. The Pt L_3_‐edge XANES spectra of Pt_1_/CN and Pt foil are shown in Figure 2b. An insignificant shift in Pt L_3_‐edge absorption was observed for Pt_1_/CN compared with Pt foil. However, Pt_1_/CN exhibits an enhanced white‐line intensity, suggesting an increased population of unoccupied d states induced by strong Pt─N interactions for Pt 5*d* electronic structure. Variations in spectral features in the EXAFS region further reflect differences in local coordination environments. A series of Pt_1_/CN samples with higher Pt single‐atom loadings is provided in Figure S3 for comparison. The corresponding *k*‐space EXAFS spectra (Figure 2c) and Fourier‐transformed *R*‐space spectra (Figure 2d) provide direct insight into the local coordination environment of Pt. For Pt_1_/CN, a single dominant peak appears at approximately 2.0 Å (phase‐uncorrected), which can be assigned to Pt─N scattering, whereas no Pt─Pt contributions are observed at longer radial distances (∼2.7 Å). The absence of Pt─Pt coordination unequivocally confirms that Pt exists in an atomically dispersed state rather than as clusters or nanoparticles. Quantitative EXAFS fitting using the DFT‐optimized distorted structural model (Figure 2e) shows excellent agreement with the experimental data, yielding a coordination environment dominated by Pt─N bonds with non‐equivalent bond lengths ranged from 2.1 to 2.7 Å. The detailed Pt─N and Pt─C coordination parameters are summarized in Table S2. In addition, wavelet‐transformed EXAFS analysis based on both *k*‐ and *R*‐space spectra provides consistent evidence for Pt coordination, revealing distinct scattering patterns corresponding to Pt─Pt interactions in Pt foil and Pt─N coordination in Pt_1_/CN, respectively (Figure 2f,g). These observations collectively suggest that the distorted and flexible g‐C_3_N_4_ framework leads to a non‐uniform Pt─N coordination geometry, which more closely resembles a Pt─N_2_‐like environment rather than an idealized symmetric planar Pt─N_4_ configuration. This finding suggests that, in mechanically compliant and distorted g‐C_3_N_4_ nanosheets, the Pt coordination environment is inherently asymmetric and cannot be adequately captured by rigid planar models. Such structural flexibility is therefore essential for accurately describing the local coordination environment of Pt single atoms. Meanwhile, the remaining coordination contributions appear less well‐defined and may be dynamically averaged, resulting in coordination heterogeneity. This heterogeneous coordination is likely one of the key factors contributing to the structural diversity of Pt single‐atom sites and the associated variability in catalytic activity.

**FIGURE 3:**
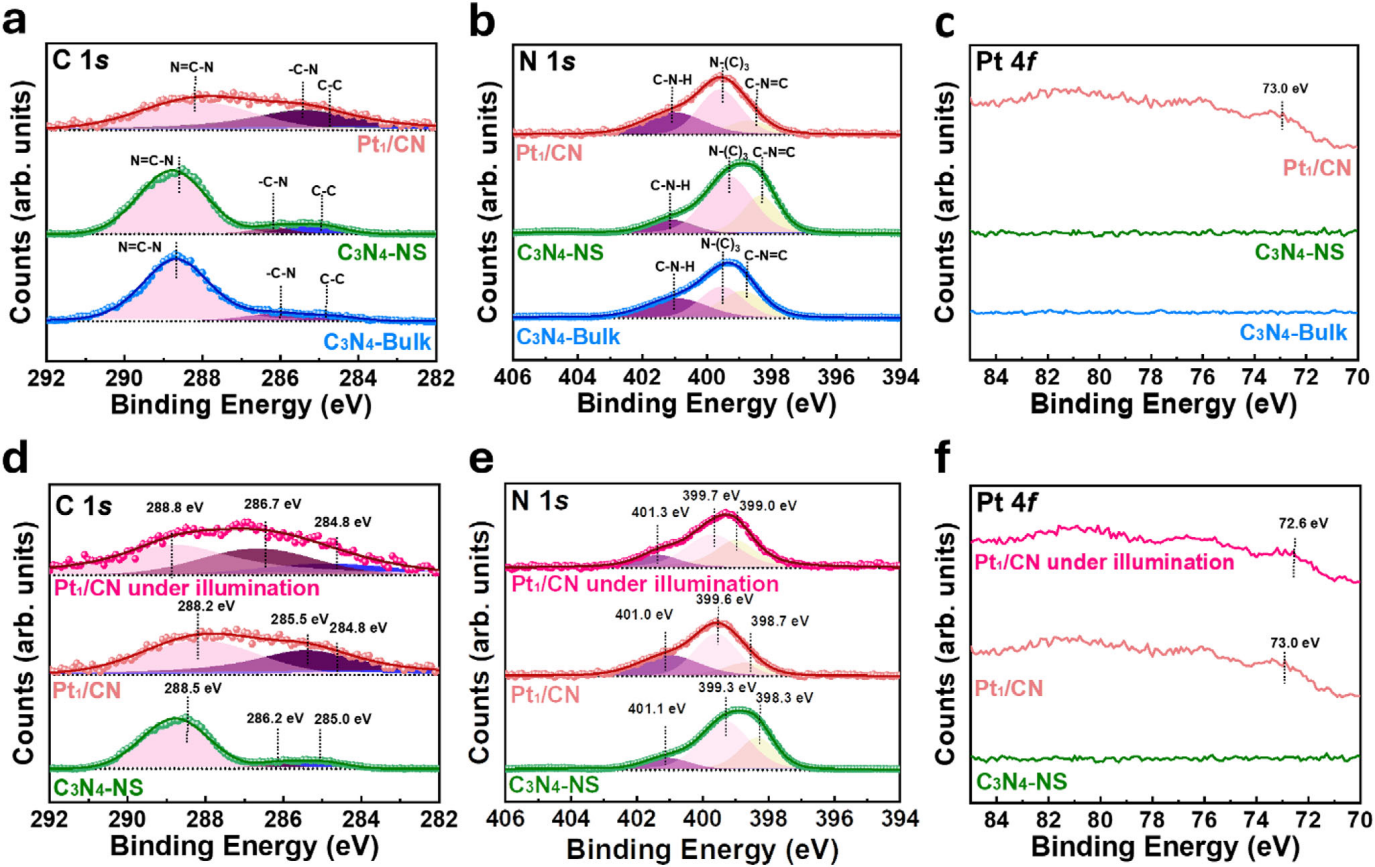
Oxidation state analysis of C_3_N_4_‐Bulk, C_3_N_4_‐NS, and Pt_1_/CN: (a) C *1s*, (b) N *1s*, and (c) Pt *4f* and in situ XPS spectra of (d) C *1s*, (e) N *1s*, and (f) Pt *4f* measured under dark condition and light irradiation for C_3_N_4_‐NS, and Pt_1_/CN.

To further elucidate the construction and electronic environments of C_3_N_4_‐Bulk, C_3_N_4_‐NS, and Pt_1_/CN, surface‐sensitive x‐ray photoelectron spectroscopy (XPS) characterizations were performed (Figure 3a–c). In the C *1s* XPS spectra of C_3_N_4_, the deconvolution typically reveals three distinct peaks. The peak observed at around 288.7 eV corresponds to the *sp* ^2^‐hybridized carbon atoms that are part of the conjugated N─C═N structure within the heptazine framework. Amino‐functional C─NH_2_ can be observed at around 285.9 eV, and the C─C bond can be observed at around 284.8 eV. In the N 1*s* XPS spectra, the deconvolution typically reveals three distinct peaks, corresponding to terminal C─N─H, *sp* ^3^ hybridized nitrogen (N─(C)_3_), and *sp* ^2^‐hybridized nitrogen (C─N═C). Upon exfoliation, the C─N═C component exhibits a downshift, indicating increased local electron density on *sp* ^2^‐hybridized nitrogen species. This behavior is attributed to enhanced structural flexibility, which enables partial reconstruction of disordered regions in bulk g‐C_3_N_4_ into a more ordered heptazine‐based framework with improved π‐conjugation and electronic delocalization. Such reconstruction of nitrogen coordination facilitates electronic coupling with Pt single atoms. Upon Pt anchoring, the weak but discernible Pt *4f* signal at approximate 73.0 eV is observed in Pt_1_/CN, which can be attributed to the ultralow Pt loading and high degree of atomic dispersion, together with the limited probing depth of XPS. A binding‐energy downshift of the C *1s* components associated with N═C─N and C─N bonds, together with an upshift of the N *1s* signals corresponding to C─N═C and N─(C)_3_ species, indicates charge redistribution within the Pt─N─C conjugated framework. Owing to the π‐conjugated nature of the N─C═N moieties, this electronic redistribution is not confined to the Pt─N interface but extends into the conjugated carbon framework. This interaction agrees the upshift of Pt *4f*_7/2_ peak (73.0 eV) relative to theoretical metallic Pt (∼71.0 eV) [37]. The transfer of photogenerated electrons was further examined by in situ XPS measurements performed in the dark and under light irradiation (Figure 3d–f). When exposed to light, all peaks in the C *1s* and N *1s* spectra shifted to higher binding energies. In opposition, the peaks of Pt *4f* spectra shifted to lower binding energies under light irradiation. This opposite shift behavior clearly demonstrates that Pt single atoms capture photogenerated electrons on Pt_1_/CN and play a crucial role in promoting the charge separation and interfacial electron transfer.

**FIGURE 4:**
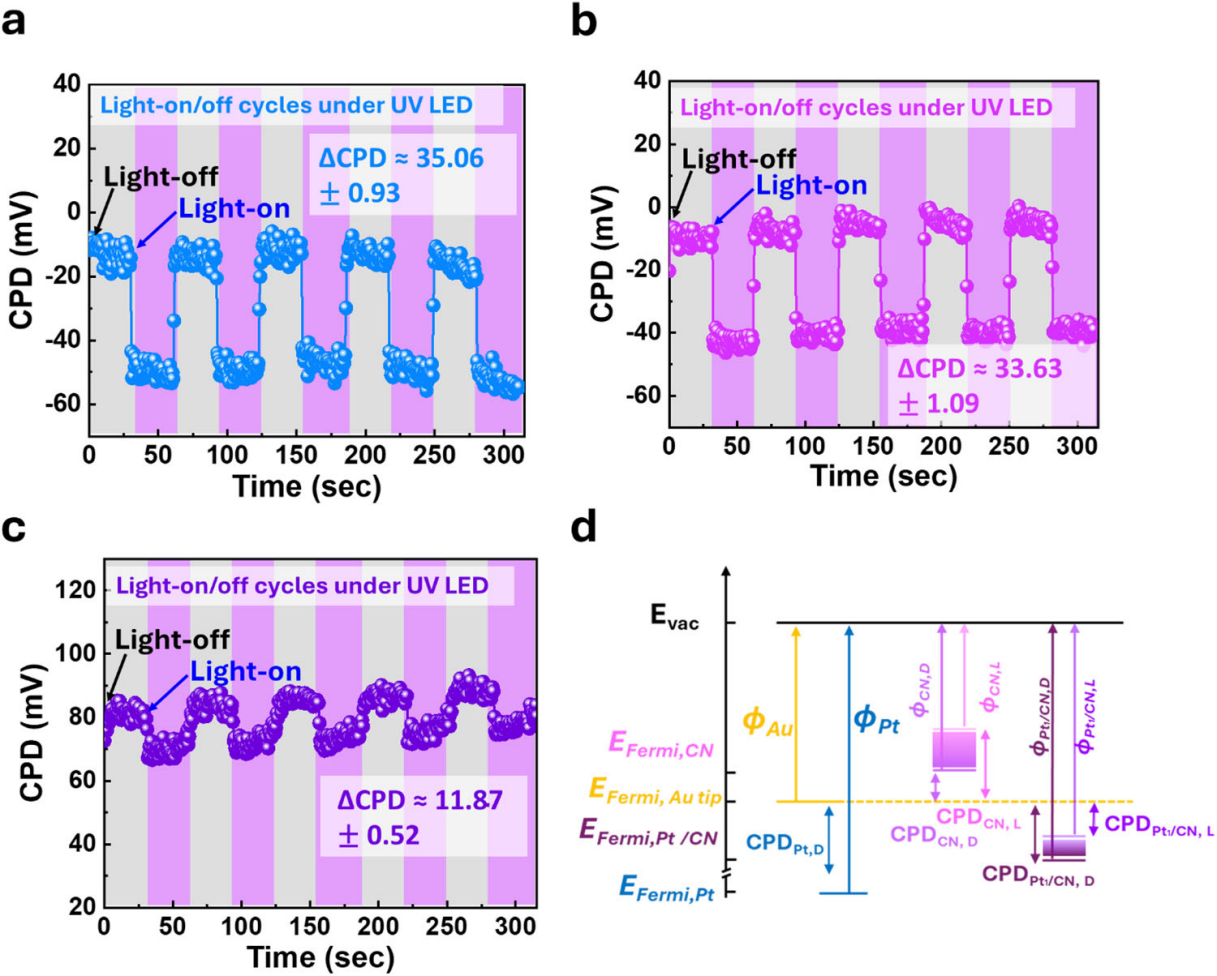
CPD changes under light on/off illumination: (a) C_3_N_4_‐Bulk, (b) C_3_N_4_‐NS, and (c) Pt_1_/C_3_N_4_. and (d) a schematic illustration of the energy‐level profile derived from Kelvin probe analysis (D: dark condition, and L: Light irradiation).

Photo assisted Kelvin probe analyzer was adopted to record the contact potential difference under illumination and in the dark for g‐C_3_N_4_ and Pt_1_/CN. During the Kelvin probe measurement, the contact potential difference (CPD) was measured using an Au probe as a reference to determine the WFs of the samples. The work function of the samples can be calculated using the equation:

(1)

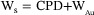

where W_S_ and W_Au_ (5.1 eV) represent the work functions of the investigated sample and Au, respectively. From Figure S4, the work function of C_3_N_4_‐Bulk was found to be 5.089 ±  0.002 eV, while a slightly lower value of 5.077 ±  0.002 eV was obtained for C_3_N_4_‐NS. This difference suggests that structural flexibility and distortion in exfoliation lead to reconstruction of the electronic structure in the nanosheets, consistent with the binding‐energy shifts observed in the XPS analysis. The incorporation of Pt single atoms led to an increase in the work function of 5.171 ±  0.001 eV. This shift indicates a significant electronic reconstruction at the interface, attributed to the strong electronic coupling between the Pt sites and the g‐C_3_N_4_ support, which is consistent with the higher electronegativity and work function of Pt. Contact potential difference under on/off light illumination provide a rapid assessment of photoactivity and directly correlate photogenerated charge carriers with the photoresponse. The CPD change (ΔCPD) is defined as follows:

(2) $$\Delta \mathrm{CPD}=\mathrm{CP}D_{\mathrm{illumination}}-\mathrm{CP}D_{\mathrm{dark}}$$

where negative CPD indicates the work function is lower than Au reference, with the opposite sign indicating the reverse. A negative ΔCPD indicates electron accumulation at the surface, while a positive ΔCPD reflects hole accumulation or electron depletion. In Figure 4a–c, the ΔCPD of Pt_1_/CN was found to be 11.87  ±  0.52 mV which is smaller than that of C_3_N_4_‐Bulk (35.06  ±  0.93 mV) and C_3_N_4_‐NS (33.63  ±  1.09 mV). The markedly reduced ΔCPD of Pt_1_/CN indicates more efficient photogenerated electron extraction and accumulation at Pt single‐atom sites. As a result, fewer free carriers are available to alter the CPD when the material is illuminated. The resulting profile of photogenerated charge behavior is illustrated in Figure 4d. Therefore, the photogenerated electrons of C_3_N_4_ could transfer to the anchored Pt single atom, promoting charge separation and thereby enhancing photoactivity.

**FIGURE 5:**
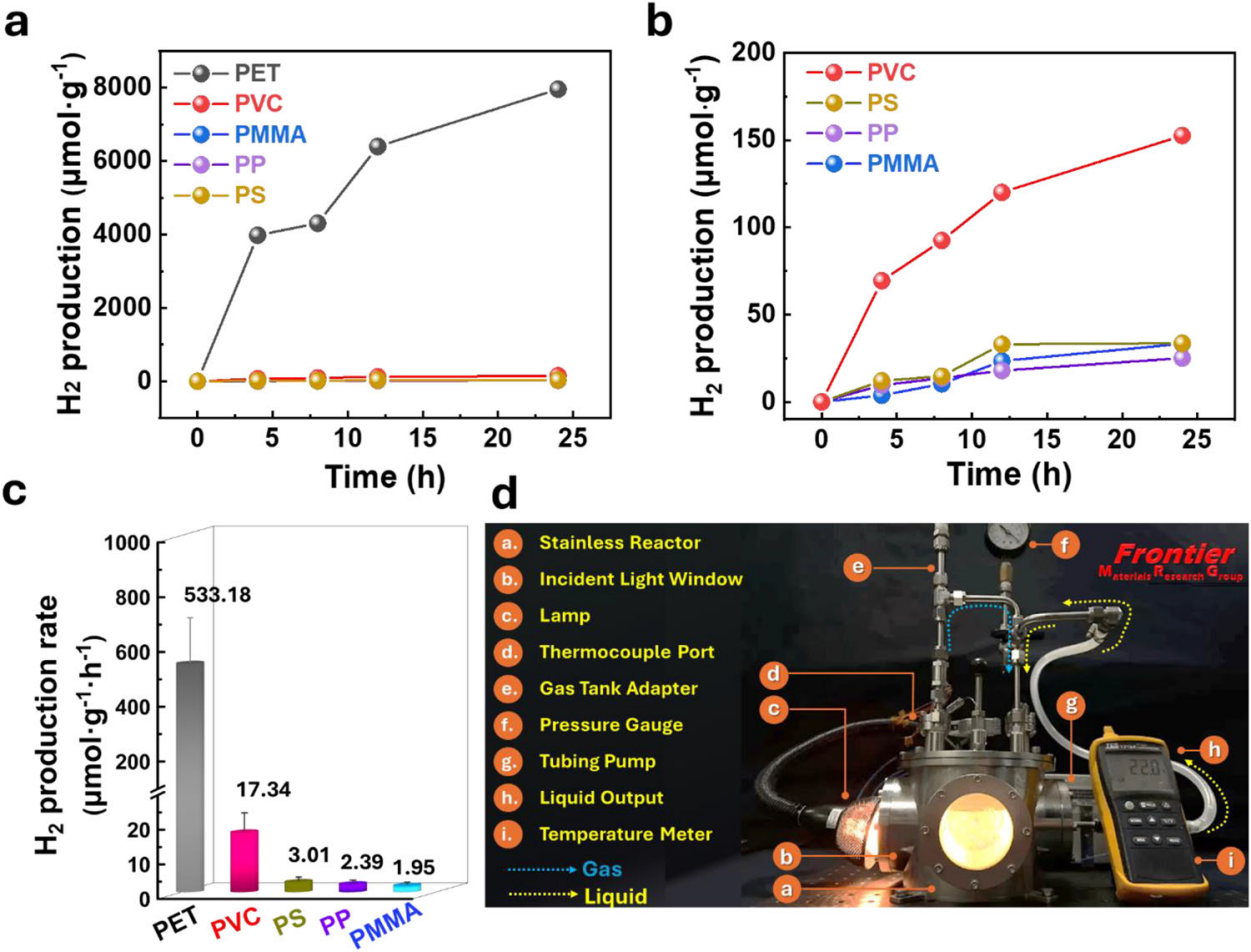
Photoreforming performance of plastics to hydrogen under Xe lamp irradiation: (a) H_2_ evolution profiles of different polymers and (b) corresponding magnified plots; (c) hydrogen production rates over Pt_1_/CN; and (d) schematic illustration of the realistically designed photoreforming reactor.

To evaluate the photocatalytic performance of Pt_1_/CN, a series of representative polymers processed into microplastic particles were subjected to photoreforming. The photoreforming of PET, PVC, PMMA, PP, and PS was systematically investigated using a sealed photocatalytic reactor equipped with Xe lamp as the light source. Table 1 lists the chemical structures, representative monomer units, and typical applications, highlighting their structural diversity and chemical complexity. Owing to their chemical inertness and limited solubility in water, all polymer substrates were subjected to alkaline pretreatment in NaOH solution (40°C, 48 h under stirring) prior to photoreforming. This pretreatment facilitates partial depolymerization of the plastics into water‐soluble oligomers or monomeric species. The resulting supernatant was subsequently used as the feedstock to initiate the photoreforming reaction, with the corresponding hydrogen yields and reaction rates presented in Figure 5a–c. The alkaline pretreatment converts solid polymers into water‐soluble species that can subsequently participate in photocatalytic oxidation. Among the tested polymers, PET exhibits the highest hydrogen production rate of 533.18 µmol·g^−1^·h^−1^ which is attributed to the presence of ester linkages that are readily hydrolyzed to generate oxygen‐rich intermediates, such as terephthalic acid and ethylene glycol. These species are more susceptible to oxidative reactions and effectively serve as oxidizable intermediates [27, 46, 47]. For PVC, the alkaline treatment induces dehydrochlorination, releasing Cl^−^ species that can be further oxidized by photogenerated holes [48], contributing to moderate hydrogen evolution activity of 17.34 µmol·g^−1^·h^−1^. In contrast, polyolefin‐based plastics with chemically inert C─C backbones exhibit strong resistance to mild alkaline hydrolysis, [49] resulting in limited formation of oxidizable intermediates and consequently low hydrogen production less than 3.0 µmol·g^−1^·h^−1^. Overall, the activity trend is governed primarily by hydrolysis accessibility and the chemical nature of the resulting intermediates of hydrolyzed plastics, further facilitating the protons reduction by photogenerated electrons on Pt_1_/CN [50, 51, 52]. The representative photoreforming performance and comparisons are summarized in Table S3. For practical advancement, the designed photoreforming reactor is demonstrated, as shown in Figure 5d. The stainless‐steel reactor is equipped with ports for light irradiation, temperature monitoring, and liquid and gas circulation. During operation, the liquid phase is continuously transported by a peristaltic pump (yellow dashed arrows) and merges with the gaseous phase (blue dashed arrows) at a junction, where the induced pressure drop enables simultaneous gas entrainment. This configuration establishes a self‐circulating flow for both liquid and gas phases without external gas pumping. As a result, the generated hydrogen is efficiently transported through the outlet adapter and accumulated in an external gas storage tank.

**TABLE 1:** Chemical structures, representative monomer units, and typical applications of major plastics.

Plastic Types	Chemical structure	Monomer	Monomer units	Applications
**PET**	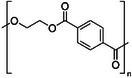	Ethylene glycol, Terephthalic acid	HO− CH_2_− CH_2_− OH, *p*‐C_6_H_4_(COOH)_2_	Disposable beverage bottles, garment fibers
**PVC**	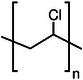	Vinyl chloride	CH_2_ = CH− Cl	Gloves, pipes, medical tubing
**PMMA**	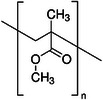	Methyl Methacrylate	CH_2_ = C(CH_3_)− COOCH_3_	Mirrors, plexiglass
**PP**	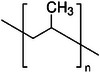	Propylene	CH_2_ = CH− CH_3_	Microwaveable containers, electronic devices, medical devices
**PS**	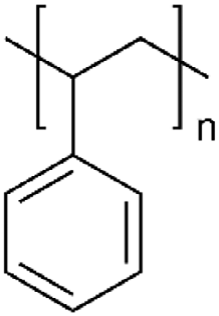	Styrene	CH_2_ = CH− C_6_H_5_	Disposable take‐away containers, electronic equipment, insulation

**FIGURE 6:**
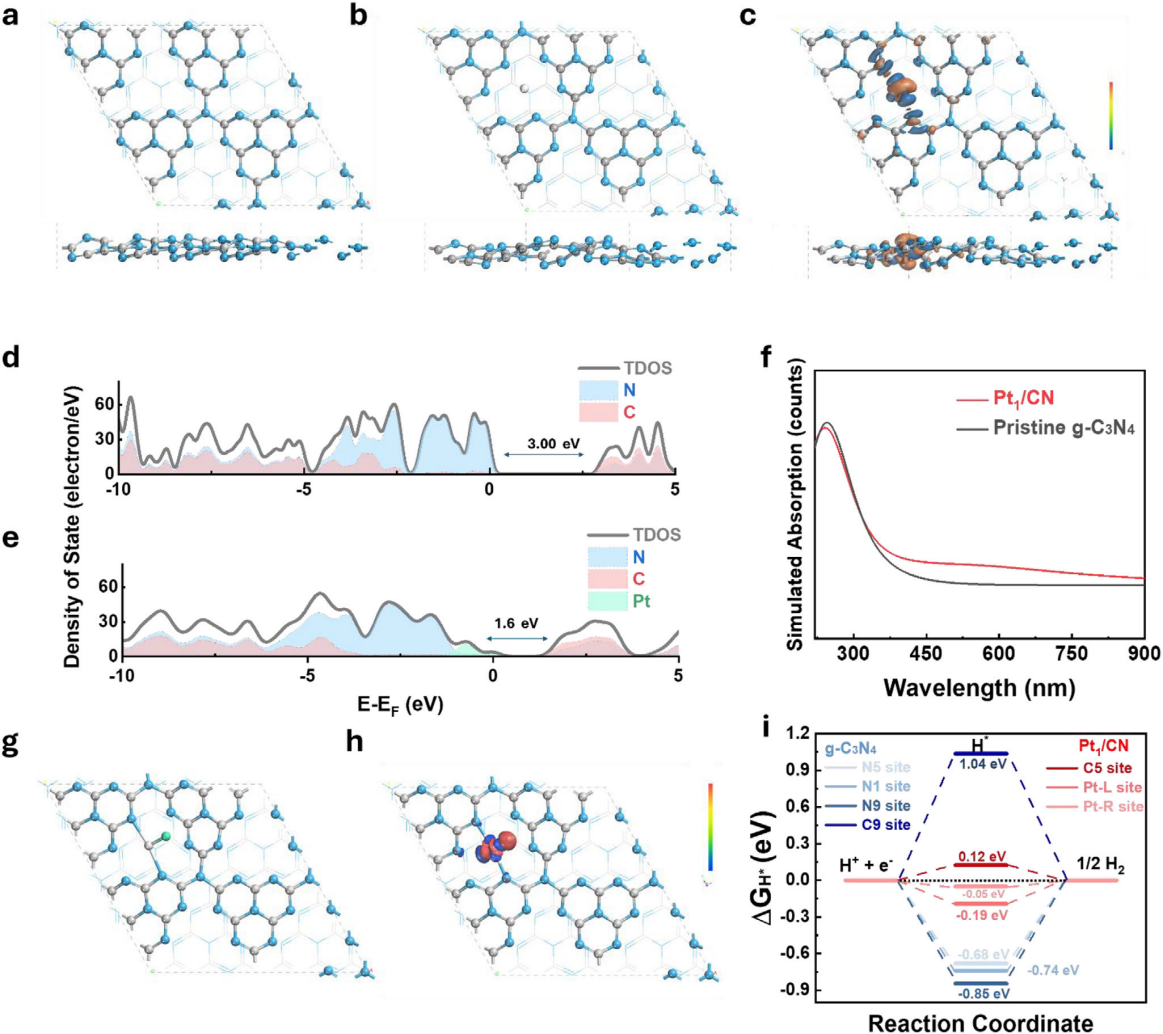
Density functional theory (DFT) analysis of g‐C_3_N_4_ and Pt_1_/CN: structural models of (a) g‐C_3_N_4_ and (b) Pt_1_/CN. (c) Electron density difference (EDD) of Pt_1_/CN shown in top and cross‐sectional views (isosurface value: 0.04). Projected density of states (PDOS) for (d) g‐C_3_N_4_ and (e) Pt_1_/CN. (f) Simulated optical absorption spectra. (g) Structural model of H adsorption on Pt_1_/CN and (h) the corresponding EDD (isosurface value: 0.05). (i) Gibbs free energy of hydrogen adsorption (ΔG_H*_) for HER on g‐C_3_N_4_ and Pt_1_/CN at different adsorption sites.

DFT calculations were employed to discover the electronic origins of the enhanced photocatalytic performance of Pt_1_/CN. In particular, the calculations focus on how the distorted g‐C_3_N_4_ nanosheet framework stabilizes Pt single atoms, modulates interfacial charge distribution, and alters the hydrogen adsorption energetics relevant to the hydrogen evolution reaction (HER). As shown in Figure 6a,b, the optimized structural models of pristine g‐C_3_N_4_ and Pt_1_/CN represent the most energetically favorable configurations. Upon Pt anchoring, a pronounced deformation of the intrinsic cavity structure is observed in the cross‐sectional view, indicating strong electronic coupling between the Pt single atom and adjacent nitrogen atoms. This structural distortion is consistent with the experimentally observed electronic modulation revealed by XPS analysis. The mapping of electron density difference in Pt_1_/CN further reveals significant electron accumulation at the Pt center (Figure 6c), accompanied by partial charge redistribution toward two neighboring nitrogen atoms, suggesting the formation of strong Pt─N interactions. In PDOS (Figure 6d,e), Pt single‐atom incorporation introduces Pt *d*‐orbital contributions near the valence band edge and substantially reshapes the electronic structure of g‐C_3_N_4_. These changes provide direct theoretical support for the experimentally observed shifts in CPD, XPS, and XANES spectra. Correspondingly, the simulated optical absorption spectra (Figure 6f) exhibit enhanced visible‐light absorption upon Pt incorporation, which is in good agreement with the experimental UV–vis results. To theoretically interpret the HER performance, the Gibbs free energy of H adsorption model was adopted. A series of geometry optimization for H adsorption on g‐C_3_N_4_ and Pt_1_/CN is revealed, shown in Figure S5. For pristine g‐C_3_N_4_, H adsorption occurs at multiple sites on the heptazine framework, exhibiting non‐uniform adsorption energetics, with thermodynamically unfavorable C sites (ΔG_H*_>0) and excessively strong N binding (ΔG_H*_<0), indicating the absence of a preferred active site. In contrast, for Pt_1_/CN, preferentially occurs at Pt‐adjacent configurations on both sides of the Pt center (Figure 6g,h), accompanied by pronounced electron transfer from the Pt atom to the adsorbed H. The calculated Gibbs free energies of hydrogen adsorption (ΔG_H*_) at these sites (Figure 6i) are close to thermoneutral values (−0.19 and −0.05 eV), approaching the ideal condition for HER. Overall, these results demonstrate that Pt single atoms stabilized within the distorted g‐C_3_N_4_ framework create electron‐rich, asymmetrically coordinated active sites that strongly facilitate proton adsorption and reduction. The presence of multiple energetically favorable H adsorption configurations near Pt centers explains the superior HER activity observed experimentally and establishes a direct structure, electronic, and photoactivity relationship for Pt_1_/CN.

## 3 Conclusion

This study demonstrates an effective photoreforming strategy for solar‐driven hydrogen production from microplastics using Pt_1_/CN nanosheets. Combined spectroscopic analyses and DFT calculations reveal that the intrinsically distorted g‐C_3_N_4_ framework stabilizes Pt single atoms in an asymmetric Pt─N coordination environment, inducing interfacial charge redistribution and efficient photogenerated electron extraction to Pt sites. Photo‐assisted Kelvin probe and in situ XPS confirm the charge separation under illumination, while DFT results show that Pt incorporation optimizes hydrogen adsorption thermodynamics with near‐thermoneutral ΔG_H*_ values. Among tested polymers, PET exhibits the highest hydrogen evolution of 533.18 µmol·g^−1^·h^−1^ due to hydrolysis‐accessible ester linkages that generate readily oxidizable intermediates. The observed activity trend correlates with hydrolysis accessibility and intermediate chemistry rather than polymer complexity. Furthermore, a practical reactor design validates the feasibility of integrating alkaline pretreatment with single‐atom photocatalysis, providing a rational pathway for sustainable hydrogen production from plastic waste.

## Author Contributions

**Ting‐Han Lin**: Methodology, Investigation, Visualization, Writing – original draft. **Yin‐Hsuan Chang**: Formal analysis, Visualization, and Writing – original draft. **Jia‐Mao Chang**: Methodology, Investigation, Visualization. **Ciao‐Yun Huang**: Methodology, Investigation. **Kai‐Chi Hsiao**: Writing – review & editing. **Kuo‐Ping Chiang**: Investigation, and Data curation. **Ying‐Han Liao**: Methodology, Investigation. **Kai‐Hsiang Hsu**: Formal analysis, Funding acquisition. **Jen‐Fu Hsu**: Formal analysis, Funding acquisition. **Ming‐Chung Wu**: Conceptualization, Supervision, Writing – review & editing, Project administration, Funding acquisition.

## Conflicts of Interest

The authors declare no conflicts of interest.

## Supporting information




**Supporting File**: advs76070‐sup‐0001‐SuppMat.docx.

## Data Availability

The data that support the findings of this study are available from the corresponding author upon reasonable request.
