# Supplementary material for: Unconstrained Coordination of Pt Single Atoms on Distorted g‐C3N4 Enables Electronic Flexibility and Enhanced Microplastics Photoreforming
Source: Adv Sci (Weinh). 2026 Jul 29;13(50):e76070. doi: 10.1002/advs.76070 (PMC13417659; doi:10.1002/advs.76070)
Supplement: Supplementary file 1 [file ADVS-13-e76070-s001.docx]

**Supporting Information**

**Unconstrained Coordination of Pt Single Atoms on Distorted g-C_3_N_4_ Enables Electronic Flexibility and Enhanced Microplastics Photoreforming**

Ting-Han Lin^1^, Yin-Hsuan Chang^2^, Jia-Mao Chang^2^, Ciao-Yun Huang^2^, Kai-Chi Hsiao^2,6^, Kuo-Ping Chiang^2^, Ying-Han Liao^2^, Kai-Hsiang Hsu^3,4^, Jen-Fu Hsu^3,5^, and Ming-Chung Wu^1,2,3,6,7*^

1.Center for Sustainability and Energy Technologies, Chang Gung University, Taoyuan 333323, Taiwan
2.Department of Chemical and Materials Engineering, College of Engineering, Chang Gung University, Taoyuan 333323, Taiwan
3.Division of Neonatology, Department of Pediatrics, Chang Gung Memorial Hospital at Linkou, Taoyuan 333423, Taiwan
4.Graduate Institute of Clinical Medical Sciences, College of Medicine, Chang Gung University, Taoyuan 333323, Taiwan
5.School of Medicine, College of Medicine, Chang Gung University, Taoyuan, 333323, Taiwan
6.Center for Heterogeneous and Innovative Post-Silicon Materials, Chang Gung University, Taoyuan 333323, Taiwan
7.Department of Materials Engineering, Ming Chi University of Technology, New Taipei City 243303, Taiwan

*Corresponding authors.

Ming-Chung Wu: e-mail, mingchungwu@cgu.edu.tw; phone, +886 3 2118800 #5771; fax, +886 3 2118800 #5324

**Experimental**

**Synthesis of catalysts**

C_3_N_4_-Bulk were initially synthesized using a high-temperature thermal polymerization method. Urea (40 g, 99.0%, Fisher Chemical) was heated to 550 °C for 3 h with a heating rate of 2 °C min^-1^. After cooling, the product was acid washed by mixing with 2.0 M HCl for 12 h and then rinsed with DI water until neutral. To obtain g-C_3_N_4_ nanosheet (C_3_N_4_-NS), exfoliation of C_3_N_4_-Bulk by ultrasonic processor (Q700 Sonicator, Qsonica, USA) using isopropanol as a solvent. For decoration of 3.0 wt% Pt single atoms, a precursor solution was prepared by dissolving PtCl_2_ (ACROS) and NaCl (SIGMA-ALDRICH) in deionized water and suspending g-C_3_N_4_ with stirring at 80 °C for 8 h. Finally, the as-obtained solid material was dried in oven at 60 °C, the final Pt atoms anchored g-C_3_N_4,_ named as Pt_1_/CN, was obtained.

**Characterization**

Crystal structures analysis was carried out by synchrotron X-ray spectroscopy (λ=1.0256 Å) at BL13A1 of the National Synchrotron Radiation Research Center (NSRRC), Hsinchu, Taiwan. The synchrotron X-ray spectra were collected from 5° to 45° of 2θ with a scan rate of 0.02°/s. The Fourier-transform infrared spectroscopy (FT-IR, Tensor 27, Bruker, Germany) was used to confirm the chemical structure of g-C_3_N_4_ in the range of 4000 to 400 cm^-1^ with a resolution of 1.0 cm^-1^. UV-VIS diffuse reflectance spectra were measured by UV-VIS spectrophotometer (V-750, JASCO, Japan). The Brunauer-Emmett-Teller (BET) surface area and Barret-Joyner-Halender (BJH) pore-size distribution were collected by surface area and porosity analyzer (ASAP 2020, Micromeritics, USA). The surface topography mappings were collected by atomic force microscopy (AFM, MultiMode, Bruker, USA). Microstructure was observed via a spherical-aberration corrected field emission transmission electron microscope (JEM-ARM200FTH, JEOL, Japan). The chemical composition and chemical state are analyzed using X-ray photoelectron spectroscopy (XPS, Thermo VG-Scientific / Sigma Probe, UK) with monochromated Al Ka radiation. The surface potential was measured by Kelvin probe force analyzer (SKP5050, KP technology, UK) equipped with a 2.0 mm-gold tip. X-ray absorption spectroscopy (XAS) and their extended X-ray absorption fine structure (EXAFS) were revealed on BL01C1 and BL 17C1 of the NSRRC, Hsinchu, Taiwan. Morphological analysis was conducted using a field-emission scanning electron microscope (FESEM, SU8010, Hitachi, Japan) at 5.0 kV.

**Computational Details**

All first-principles calculations were performed using the CASTEP module using BIOVIA Materials Studio 2024, based on density functional theory (DFT). The distorted structural model of pristine heptazine based g-C_3_N_4_ model ^[1]^ was adopted. Prior to production calculations, convergence tests were conducted to determine suitable plane-wave cutoff energy and k-point sampling. A 2×2×1 supercell of heptazine based g-C_3_N_4_ model containing two atomic layers and a 10.0 Å vacuum layer along the z-direction was constructed to minimize periodic image interactions and to accommodate single Pt atom decoration and hydrogen adsorption. Geometry optimization was carried out using the Limited-memory Broyden–Fletcher–Goldfarb–Shanno (LBFGS) algorithm. The bottom atomic layer of the g-C_3_N_4_ slab was constrained to simulate a supported surface. The exchange-correlation functional was treated within the Generalized Gradient Approximation (GGA) using the Perdew-Burke-Ernzerhof (PBE) formulation. On-the-fly generated (OTFG) ultrasoft pseudopotentials were employed to describe electron-ion interactions. A plane-wave cutoff energy of 550.0 eV and a Monkhorst-Pack k-point mesh of 1 × 1 × 1 were used during optimization due to the large supercell size and surface slab geometry, which was validated by convergence testing. After geometry convergence, the electronic band structure was calculated along standard high-symmetry directions in the Brillouin zone. The projected density of states (PDOS) was computed using a denser k-point sampling (2×2×1) and a Gaussian smearing width of 0.2 eV to ensure a smooth spectrum. The electron density difference (EDD) was calculated to visualize charge redistribution by subtracting the charge densities of the isolated g-C_3_N_4_ slab and Pt atom from that of the combined Pt_1_/CN system. To study the catalytic activity for the HER, a single hydrogen atom was adsorbed on the Pt_1_/CN surface. The adsorption energy (∆$\text{E}_{\text{abs}}$) was evaluated using the equation:

∆$E_{abs}{=E}_{slab+H}-E_{slab}- \frac{1}{2}E_{H2}$	(S1)

The Gibbs free energy of hydrogen adsorption (ΔG_H_) was then obtained by including zero-point energy (E_ZPE_) and entropy corrections:

${\Delta G}_{H}=\Delta E_{abs}+ \Delta E_{ZPE}-T\Delta S_{H}$	(S2)

where $\Delta E_{ZPE}$is the change of the zero-point energy between adsorbed state and gas phase. The $\Delta S_{H}$was entropy change of H adsorption at T=300K. Therefore, the expression ^[2]^ can be simplified as:

$\Delta G=\Delta E_{abs}+$0.24 eV	(S3)

**Photoreforming Measurements**

The photoactivity of the catalysts was studied using a sealed reactor at ambient temperature using the pre-treatment PET solution. The irradiation source was a solar simulator (Oriel LCS-100TM, Newport, US), featuring a 100 W Xe lamp. In a typical experiment, 50.0 ml of PET pretreatment (in 5.0 M NaOH) and 50.0 mg of photocatalyst was added into the reaction cell. After completely sealed with rubber septa, the reactor was purged for 30 min by N_2_ gas (50.0 ml∙min^-1^) in the dark and stirred at 400 rpm. The peak positions of gas components and appearance of oxygen and nitrogen in the reactor were checked by GC analyses before starting the photoreaction. Finally, the reactor was irradiated using solar simulator with N_2_, constant temperature was kept at 25$\pm$2°C and stirred at 400 rpm during irradiation. For quantity of the produced H_2_, the representative H_2_ production rate is defined as:

H_2_ production rate (μmol g^-1^ h^-1^) = $\frac{\text{H}\text{2}\text{ production at 12 h (}\text{μmol}\text{) }}{\text{mass of catalyst (g) ×12 (h)}}$	(S4)

**Figure S1.** FESEM images of **(a)** C_3_N_4_-Bulk, **(b)** C_3_N_4_-NS, and **(c)** Pt_1_/CN; Insets: corresponding low-magnification TEM images.

**Figure S2.** BET analysis of fabricated C_3_N_4_-Bulk and C_3_N_4_-NS.

**Figure S3.** Pt L_3_-edge X-ray absorption spectroscopy (XAS) of samples with different Pt loadings. **(a)** XANES spectra. **(b)** k-space EXAFS spectra. **(c)** Fourier-transformed EXAFS spectra in R space. **(d-g)** Wavelet-transform EXAFS maps of **(d)** Pt foil and samples with Pt loadings of **(e)** 1.0 wt%, **(f)** 3.0 wt%, and **(g)** 5.0 wt%.

**Figure S4**. Contact potential difference of **(a)** C_3_N_4_-Bulk, **(b)** C_3_N_4_-NS and **(c)** Pt_1_/CN.

**Figure S5**. DFT-optimized H adsorption structures of **(a)** g-C_3_N_4_ and **(b)** Pt_1_/CN, together with **(c)** the corresponding atomic models showing atom-number labeling.

**Table S1.** The specific surface area, pore volume, and pore size of C_3_N_4_-Bulk and C_3_N_4_-NS

| **Sample** | ***S*_BET_ (m^2^⸳g^-1^)** | **Pore Volume (cm^3^⸳g^-1^)** | **Pore Size (nm)** |
| --- | --- | --- | --- |
| C_3_N_4_-Bulk | 72.28 | 0.42 | 23.21 |
| C_3_N_4_-NS | 77.77 | 0.51 | 24.30 |

**Table S2.** EXAFS fitting parameters for Pt_1_/CN derived from the Pt L_3_-edge EXAFS spectra

| **Path** | **CN** | **R (Å)** | **σ^2^ (10^3^ Å^2^)** | **ΔE_0_ (eV)** | **R-factor** |
| --- | --- | --- | --- | --- | --- |
| Pt-N_57, 74_ | 2.0 | 2.09 | 0.02 | 16.99 | 0.02 |
| Pt-N_72, 86_ | 2.0 | 2.51 | 0.30 | 3.21 |  |
| Pt-N_17_ | 1.0 | 2.67 | 6.13 | 14.83 |  |
| Pt-C_19_ | 1.0 | 2.72 | 0.59 | 14.83 |  |
| Pt-N_59_ | 1.0 | 2.77 | 8.29 | 14.83 |  |
| Pt-C_80_ | 1.0 | 2.78 | 6.36 | 14.83 |  |
| Pt-C_88_ | 1.0 | 2.82 | 7.83 | 14.83 |  |

**Table S3**. Summary of representative photocatalytic performance for plastic photoreforming to hydrogen over Pt loaded C_3_N_4_ systems.

| Photocatalysts | Plastic type | Pretreatment | Reaction condition | H_2_ production Rate (x hour)**^a^** (μmol g_cat_^-1^ h^-1^) | Ref. |
| --- | --- | --- | --- | --- | --- |
| Pt_1_/CN | PET (Bottle) | 1.0 mg mL^-1^ Plastics in NaOH (5.0 M) at 40^o^C for 48h | 1.0 mg_cat._ mL^-1^  100W Xe Lamp | 533.18 (12h) | This study |
|  | PVC (Plastic Wrap) |  |  | 17.34 |  |
|  | PS (Petri Dish) |  |  | 3.01 |  |
|  | PP (Straw) |  |  | 2.39 |  |
|  | PMMA (Container) |  |  | 1.95 |  |
| MCN (Pt 3wt.%) | PET Powder | 10.0 mg mL^-1^ Plastics in NaOH 5.0M at 70 ^o^C for 72h | 1.0 mg_cat._ mL^-1^  100 W Xe lamp with an AM 1.5G filter | 7,330 (6h) | ^[3]^ |
| ^NCN^*g*-C_3_N_4_\|Pt | PE | 27.3mg mL^-1^ Plastic in HNO_3_ (6 wt%) at 180 °C hydrothermal reaction, 4 h | 2.0 mg_cat._ mL^-1^/2.0 mL of 10.0 mg mL^-1^ succinic acid in 0.1 M HNO_3_.  100 W Xe lamp with an AM 1.5G filter | 137 (72h) | ^[4]^ |
| Pt/g-C_3_N_4_ | PET granule | 1.0 mg mL^-1^ Plastic/0.40 mg mL^-1^ Binuclear zinc complex in NaOH (0.1 M) at 60 °C for 48 h | 1.0 mg_cat._ mL^-1^  100W Xe lamp with a 420 nm cutoff filter | 400 (4h) | ^[5]^ |
| Pt_1_/P doped CN (PCN) | PET granules | 50.0 mg mL^-1^ Plastics in 5.0M KOH at 70^o^C for 24h | 0.4 mg_cat._ mL^-1^  300 W Xe lamp with AM 1.5G filter. | 2,722.8 (n/a) | ^[6]^ |
| Pt SA/BCN | PLA plastic powder | n/a | 1.25 mg_cat._ mL^-1^ in seawater  300 W Xe Lamp | 993 (12h) | ^[7]^ |

**Note**: mg_cat_. denotes the weight of used catalyst; **a** hydrogen evolution rates were calculated based on the total H₂ produced over a reaction duration of “X”; n/a indicates that the corresponding data are not available.

**Reference**

[1]	J. J. Wang, D. Hao, J. H. Ye, N. Umezawa, *Chem Mater* **2017**, 29, 2694.

[2]	X. J. Li, S. Y. Zhao, X. G. Duan, H. Y. Zhang, S. Z. Yang, P. P. Zhang, S. P. Jiang, S. M. Liu, H. Q. Sun, S. B. Wang, *Appl Catal B-Environ* **2021**, 283.

[3]	T. K. A. Nguyen, T. Trần-Phú, X. M. C. Ta, T. N. Truong, J. Leverett, R. Daiyan, R. Amal, A. Tricoli, *Small Methods* **2024**, 8, 2300427.

[4]	C. M. Pichler, S. Bhattacharjee, M. Rahaman, T. Uekert, E. Reisner, *ACS Catal.* **2021**, 11, 9159.

[5]	M. Li, S. Zhang, *ACS Catal.* **2024**, 14, 2949.

[6]	X. Bao, M. Li, Y. Xie, X. Luo, F. Tong, X. Liang, D. Xiao, Z. Wang, *J Colloid Interf Sci* **2026**, 716, 140317.

[7]	Z. Ya, M. Li, D. Xu, H. Wang, S. Zhang, *ACS Nano* **2025**, 19, 16011.
